# Effect of Site-Specific Functionalization on the Shape of Nonspherical Block Copolymer Particles

**DOI:** 10.3390/polym12122804

**Published:** 2020-11-26

**Authors:** Jaeman J. Shin

**Affiliations:** Department of Chemical and Biomolecular Engineering, Korea Advanced Institute of Science and Technology (KAIST), Daejeon 34141, Korea; jmshin90@gmail.com or jmshin90@ucsb.edu

**Keywords:** block copolymer, particle shape, site-specific functionalization, thiol-ene

## Abstract

Shape-anisotropic polymeric colloids having chemically distinct compartments are promising materials, however, introducing site-specific surface functionality to block copolymer (BCP) particles has not yet been actively investigated. The current contribution demonstrates the selective surface functionalization of nanostructured, ellipsoidal polystyrene-b-polybutadiene (PS-b-PB) particle and investigate their effects on the particle shape. Photo-induced thiol-ene click reaction was used as a selective functionalization chemistry for modifying the PB block, which was achieved by controlling the feed ratio of functional thiols to the double bonds in PB. Importantly, the controlled particle elongation was observed as a function of the degree of PB functionalization. Such an increase in the aspect ratio is attributed to the (i) increased incompatibility of the PS and modified PB block and (ii) the reduced surface tension between the particles and surrounding aqueous medium, both of which contributes to the further elongation of ellipsoids. Further tunability of the elongation behavior of ellipsoids was further demonstrated by controlling the particle size and chemical structure of functional thiols, showing the versatility of this approach for controlling the particle shape. Finally, the utility of surface functionality was demonstrated by the facile complexation of fluorescent dye on the modified surface of the particle via favorable interaction, which showed stable fluorescence and colloidal dispersity.

## 1. Introduction

Surface functionalization of polymeric colloids has been an area of interest for both polymer chemists and colloidal scientists, relating to many important applications including colloidal assembly [1,2,3], fluorescent probe [4], drug delivery [5], and stimuli-responsive smart particles [6]. Several strategies have been developed for generating polymeric nano- and microparticles with desired surface functionality. Variation among emulsion/dispersion polymerization techniques with the copolymerization of functional monomers allowed epoxy, hydroxyl, amine and carboxylic acid functionalized particles [7,8,9,10]. Furthermore, the secondary transformation of these surface functional groups to clickable moiety allowed the efficient grafting of biomolecules [11,12]. However, it remains challenging to achieve the site-specific surface functionalization of nonspherical polymeric colloids via simple and facile functionalization chemistry, in contrast to the reported works where surface modification was applied to the entire surface of spherical particles. While compartmentalized polymer particles served as a good platform for localized surface functionalization, the majority of the works were limited to relatively simple Janus or dumbbell particles [13,14,15,16], and investigation of the site-specific functionalization of polymer particles with a more advanced morphology and shape have not been actively investigated.

Recently, block copolymer (BCP) particles received great attention as an advanced polymeric colloid exhibiting several promising features that are advantageous for localized, selective functionalization. Utilizing the self-assembly of BCP confined in evaporative emulsion droplet, nanostructured particles with a well defined shape and size can be generated by simple solvent evaporation [17,18,19]. Moreover, spontaneous deformation into spheroids (prolate and oblate ellipsoids) can be easily achieved based on the deformability of the particle interface [20,21,22]. The key idea is to achieve the perpendicular alignment of BCP to the particle surface, either by engineering the interfacial interaction via functional surfactants [23,24,25,26,27,28,29,30] or controlling the self-assembly kinetics [31,32]. Driven by the simplicity and cost-effectiveness of such an approach for generating nonspherical polymer colloids having periodic nanostructures, significant efforts were devoted to achieving the precise controllability particle size and shape [33,34,35], expanding the scope of the accessible polymers [36,37,38,39] and generating non-typical shapes [40,41]. Nevertheless, the site-specific chemical functionalization of the surface of BCP particles is less realized [42,43] in contrast to the BCP bulk and thin film [44], and the currently established functionalization approaches relied on mediating the physical interaction of each building block during the co-assembly of multicomponents in emulsion [45,46,47,48,49].

Here, the site-specific surface functionalization of nanostructured BCP particle was investigated, with a particular focus on the effect of functionalization on the particle shape. Thiol-ene click reaction is utilized as a functionalization chemistry for the selective modification of a PB block of poly(styrene-*b*-butadiene) (PS-*b*-PB) prolate ellipsoid, using mercaptopropionic acid as a model functional thiol. The selective functionalization of PB domains on the particle surface was confirmed via spectroscopic analysis, and the degree of PB modification was controllable by varying the feed ratio of thiols to the double bonds in the PB domain. Particle elongation was observed upon PB functionalization as a function of the degree of modification, to result in the controllable aspect ratio (AR) of the ellipsoid. This is due to the increased incompatibility of PS and modified PB block and lowered surface tension of the particles, both of which contribute to the further elongation of ellipsoids. The tunability of the elongation of ellipsoids was further demonstrated by controlling the particle size and chemical structure of functional thiols, showing the versatility of the approach for tuning particle shape. Finally, the carboxylic acid functional group on the particle surface was utilized for the facile and stable binding of fluorescent dye.

## 2. Experimental

### 2.1. Materials

Symmetric poly(styrene-*b*-butadiene) (PS_34k_-*b*-PB_25k_, 1,4 addition rich > 90%) BCP was purchased from Polymer Source, Inc (Dorval, QC, Canada) (subscripts indicate the number-average molecular weight (Mn) of each block). Sodium dodecyl sulfate (SDS). Mercaptopropionic acid (MPA), pentaerythritol tetrakis(3-mercaptopropionate) (PETMP), 1H, 1H, 2H, 2H perfluorodecanethiol (PFDT), (2-mercaptoethyl)trimethylsilane (MTMS), and benzophenone (BzP) were purchased from Sigma Aldrich (St. Louis, MO, USA). Toluene and tetrahydrofuran (THF) were purchased from Samchun Chemical (Seoul, Korea).

### 2.2. Characterization

Proton nuclear magnetic resonance spectra (^1^H NMR) were recorded on a Bruker NMR spectroscopy (Bruker Instruments, Karlsruhe, Germany) system operating at 300 MHz. Fourier-transform infrared spectroscopy (FT-IR) measurement was performed on an Bruker Alpha FT-IR spectrometer (Bruker Instruments, Karlsruhe, Germany). FT-IR samples were prepared by concentrating the particles by centrifugation followed by drying the aqueous phase in vacuum at 40 °C, yielding slight white-yellow powders. NMR samples were prepared by dissolving these powders in deuterated chloroform (CDCl_3_). Field-emission scanning electron microscopy (FE-SEM, Nova230, Japan), and transmission electron microscopy (TEM) (JEOL 2000FX, Japan) were used to observe the particle shape and internal morphology. For TEM samples, the PB domains in the particle suspension was stained with 0.2 wt% osmium tetraoxide (OsO_4_) followed by drop-casting onto copper grids coated with 20–30 nm thick carbon and dried in air. For SEM samples, the particle suspension was drop-casted onto silicon wafers, which were then dried and coated with platinum (Pt) in 3 nm thickness. Fluorescent microscopy (Leica, Germany) was performed with a 533 nm excitation wavelength.

### 2.3. Preparation of PS_34k_-b-PB_25k_ Ellipsoids via Membrane Emulsification

A solution containing 10 mg/mL of PS_34k_-*b*-PB_25k_ in toluene (3 mL) was emulsified in a continuous phase containing 5 mg/mL SDS in deionized (DI) water (60 mL) using a Shirasu porous glass (SPG) membrane device [33]. Monodisperse emulsion droplets of PS-*b*-PB were generated by passing the organic phase through the SPG membrane. To control the droplet size, membranes with various pore sizes (*d_pore_*) including 0.5, 1.1 and 2.1 were employed. After emulsification, the interfacial area between emulsion and air was kept constant (A_emul/air_ = 26.4 cm^2^), so that the evaporation rate was fixed to the ellipsoid-forming condition [31].

### 2.4. Modification of PB domain of the Ellipsoidal Particles via Thiol-Ene Photochemistry

To the 3 mL of PS_34k_-*b*-PB_25k_ particle suspension (0.5 mg/mL, 1 equiv of C=C bonds) that were produced from *d_pore_* = 0.5 μm, designated amount (*r*, mole ratio of thiol to the olefin in PB) of THF solution containing functional thiol (10 mg/mL) was added. Twenty-two microliters (0.01 equiv) of BzP photoinitiator solution in THF (1 mg/mL) and an additional 0.4 mL of THF were added to achieve the overall 15/85 (*v*/*v*) ratio of THF to water. Mixture solution was stirred for 30 min before being transferred to a quartz glass cell and was exposed to a UV lamp (λ = 365 nm) for a designated time under stirring. Then, the solution was transferred to open vial and THF was evaporated under gentle stirring. Afterwards, the sample was repeatedly washed with DI water via centrifugation (5 min, 10,000 rpm) for more than 3 times to remove surfactants, unreacted thiols, and photoinitiators.

## 3. Results and Discussion

**Scheme 1:**
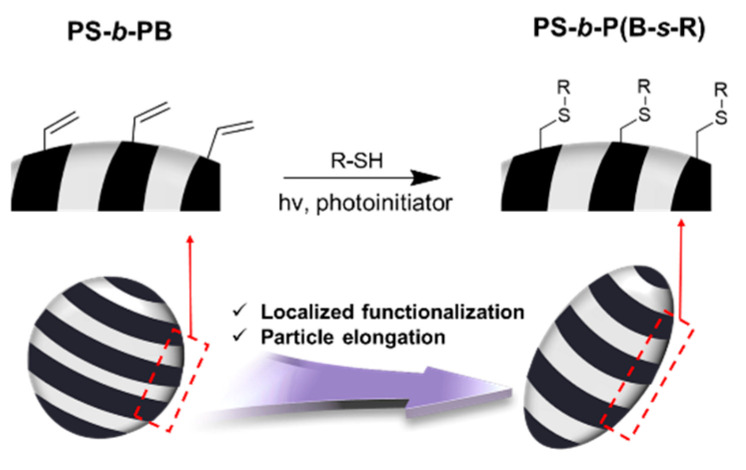
Schematic illustration showing the site-specific chemical modification of PB domains on the surface of the PS_34k_-*b*-PB_25k_ ellipsoid via radical thiol-ene photochemistry.

Scheme 1 shows the experimental system in this work, illustrating the localized surface modification of PS_34k_-*b*-PB_25k_ particles and corresponding changes in the particle shape. For the successful site-specific functionalization of BCP particles, the following key aspects were addressed. First, the precursor particle for surface modification requires both PS and PB blocks exposed to the surface, thus offering olefinic sites in PB domains for modification while the PS block remains inert. Therefore, the ellipsoidal particle with a perpendicular orientation of the PS_34k_-*b*-PB_25k_ domain was achieved via controlling the solvent evaporation rate of emulsion as reported in the previous work [31]. Second, efficient chemistry suitable for the modifying PB domain was needed. Photoinitiated radical thiol-ene click chemistry was selected as a robust and modular reaction [50,51,52], to benefit from the high reactivity of thiols in concert with the fast ligation of functional molecules. Furthermore, the feed ratio of thiols to olefinic sites in the PB block (*r =* [R-SH]/[C=C]) was adjusted for the systematic control on the extent of PB modification. Also, functional thiols with different chemical structures were employed for the investigation of their effect on the particle shape and morphology. Third, the efficient diffusion/transport of functional thiols to the particle through the aqueous phase is necessary. For this purpose, functional thiols were dissolved in THF and added to the aqueous dispersion containing PS_34k_-*b*-PB_25k_ precursor particles before the reaction by exposure to UV light. THF was used as a thiol-directing solvent as it is miscible in water and a good solvent for both PS and PB. Additionally, THF can serve as an annealing solvent for the reconstruction of particle shape and morphology accompanied by the chemical modification.

To investigate the selective surface modification process via thiol-ene chemistry, PS_34k_-*b*-PB_25k_ particles produced by membrane emulsification from *d_pore_* = 0.5 μm and MPA were used as a model precursor particle and functional thiol. A varying amount of MPA (*r* = 0.1, 0.2, 0.5) and BzP photoinitiator in THF was added before reacting via UV irradiation for 1 h. A notable difference was observed during the purification process via centrifugation, where neat PS_34k_-*b*-PB_25k_ particles floated on the top while particles modified with MPA settled on the bottom upon centrifugation (Figure S1). This observation indicated the substantial conjugation of MPAs (*ρ_MPA_* = 1.22 g/mL) on PS_34k_-*b*-PB_25k_ particles having a density slightly smaller than the aqueous surrounding (*ρ_PS-b-PB_* = 0.97 g/mL).

**Figure 1:**
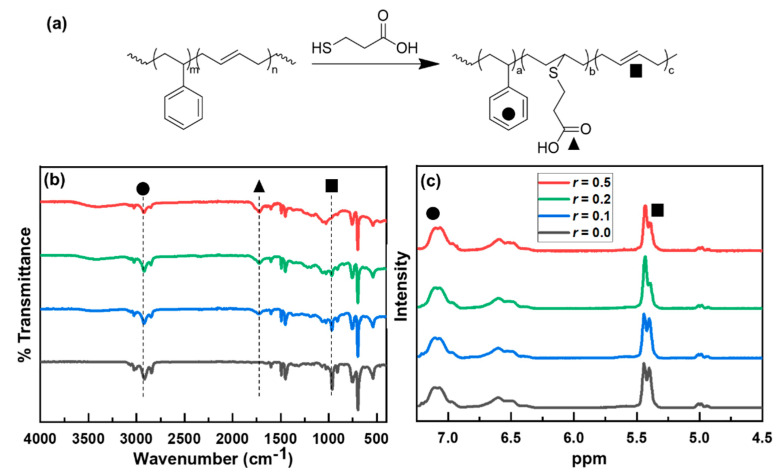
(**a**) Reaction scheme for the selective PB modification for PS_34k_-*b*-PB_25k_ particles by mercaptopropionic acid (MPA); (**b**) FT-IR spectra of particles for *r* = 0.0, 0.1, 0.2, 0.5; (**c**) ^1^H NMR spectra of pristine PS_34k_-*b*-PB_25k_ particles (*r* = 0, black) and MPA-modified particles with a varying MPA feed ratio (*r* = 0.1, blue; *r* = 0.2, green; *r* = 0.5, red). The reaction time was fixed to 1 h.

To further confirm the selective modification of the PB domain, characterization via infrared spectroscopy was performed (Figure 1a). Comparing the FT-IR spectra of pristine PS_34k_-*b*-PB_25k_ and MPA-modified particles, two major differences were observed. First, a decrease in the peak intensity near 908 cm^−1^ corresponding to the alkene =C–H bend (blue) was observed (square), reflecting that the double bonds in the PB domain participated in the thiol-ene functionalization. Second, a new signal appeared in 1722 cm^−1^ corresponding to the carbonyl (C=O, purple) stretch of the carbonyl peak (triangle), which is expected to arise from the presence of a carboxyl acid group. An aromatic C–H signal from the PS block in the range of 2800–3000 cm^−1^ (green) remained intact (circle). A more clear visualization by the normalization of peaks to the aromatic C–H signal showed a gradual increase in carbonyl (C=O) peak intensity near 1722 cm^−1^ (Figure S2), reflecting the increasing degree of PB modification as a function of *r*.

Similarly, the gradual decrease in peak intensity for vinyl protons (square) compared to the aromatic proton peaks (circle) was observed in ^1^H NMR as a function of *r* (Figure 1b). For further quantitative analysis on the degree of modification for each particle (*r* = 0.0, 0.1, 0.2, 0.5), the integration value of aromatic signals (6.4–7.2 ppm) and the signals from vinylic protons in PB (4.9–5.5 ppm) were compared (Figure S3). The C=C conversion increased as a function of *r*, with good correspondence to each feed ratio (0.09 for *r* = 0.1 and 0.22 for *r* = 0.2), except for the case of *r* = 0.5 where the actual conversion (=0.36) was lower than the feed ratio. This discrepancy was expected to occur due to the limited accessibility of MPAs to the interior of the PB domains. As another control experiment, the MPA modification of onion-like particles showed much less functionalization with no changes in the particle shape and domain size (Figure S4), reflecting that the majority of functionalization occurs at the PB domain on the particle surface and there is an upper limit on the accessibility of function thiols to the interior of the particle due to the low solubility of MPAs in the polymer domains.

**Figure 2:**
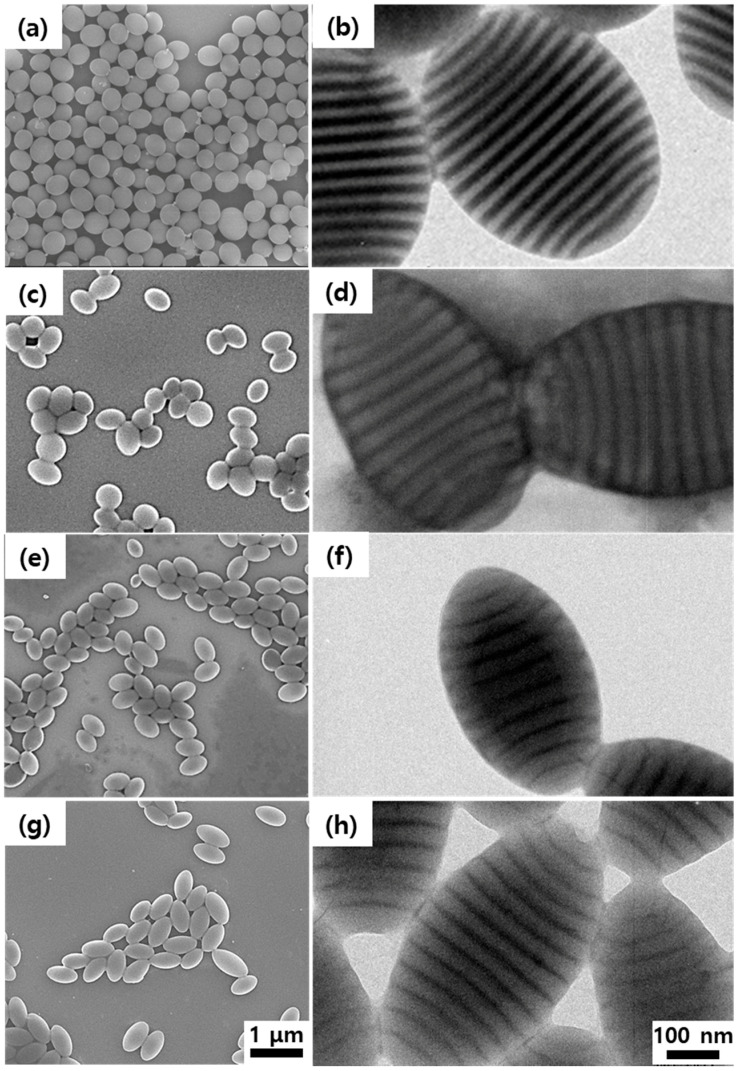
SEM and TEM images of (**a**,**b**) as-prepared PS_34k_-*b*-PB_25k_ particles, and after modification with MPA with (**c**,**d**) *r* = 0.1, (**e**,**f**) *r* = 0.2, (**g**,**h**) *r* = 0.5. PB domains were stained with OsO_4_ before TEM imaging.

Figure 2 shows the structural characterization of pristine PS_34k_-*b*-PB_25k_ particles and MPA-modified particles using SEM and TEM. Uniform-sized precursor PS_34k_-*b*-PB_25k_ particles was obtained showing a slight ellipsoidal shape with axially stacked lamellae. (Figure 2a,b). Interestingly, the elongation of PS_34k_-*b*-PB_25k_ particles was observed after treating with MPA, while retaining the axially stacked lamellae structure and the particle size uniformity (Figure 2c–h). To further confirm the localized conjugation of MPAs only at the PB domain surface, the particle was treated with Au precursor (HAuCl_4_∙3H_2_O) and compared with an OsO_4_ stained sample under TEM (Figure S5). Identical dark domains in the striped lamellae pattern for both cases reflects that a notable amount of unreacted alkene still remained to be stained with OsO_4_, and the PB domain was modified since metal salts favorably interact with functionalized carboxylic acid groups at the PB domain surface [53]. Under exposure to the TEM electron beam, a selective growth of Au nanoparticles within the MPA-modified PB domain occurs through in situ reduction [54].

Detailed analysis on the structural difference of the particles upon MPA modification was performed by statistical measurements on the characteristic lengths including domain spacing, major axis (*L*), minor axis (*S*), and AR, obtained by measuring more than 100 particles using ImageJ and summarized in Table 1. The increase in the average *L_0_* upon MPA modification is attributed to the additional volume input from MPA conjugation. While the increase in *L*_0_ was not significant, the domain thickness of gray (*d_G_*) and dark layers (*d_D_*) in TEM images showed notable difference where *d_D_* decreased from 19, to 16, and to 14 nm. Such difference is presumably due to a lesser amount of remaining double bonds in the PB domain being unable to be stained with OsO_4_ with a higher extent of modification by MPA. Moreover, a sharp increase in AR was observed from 1.20 to 1.56 upon MPA modification with *r* = 0.1 (Figure 2c,d), and AR values saturated to 1.75 and 1.78 with a further increase in *r* to 0.2 and 0.5 (Figure 2e–h). Such an increase is remarkable, as it is relative to the slight increase in *L*_0_. No further elongation in the particle shape was observed upon the additional reaction by UV irradiation for longer times (Figure S6), suggesting that the modification of the PB domain was completed before 1 h. Furthermore, the saturation behavior of AR supports the limited C=C conversion observed in the ^1^H NMR analysis, even at a higher feed ratio (*r* = 0.5).

The elongation of ellipsoidal particles can be understood based on the previous literature describing the elongation behavior of BCP ellipsoids as a counterbalance of (i) the interfacial energy between two blocks of BCP, (ii) entropic penalty associated with chain stretching/bending upon particle elongation, and (iii) the surface energy between the particle and surrounding [23,24,34,35]. Particles elongate until the interfacial energy and entropy of chain stretching is minimized to satisfy the commensurability condition, while the energetic cost of the increasing surface area between the particle and the surrounding opposes the shape deformation. The counterbalance of these contributions determines the AR. Based on this principle, a notable increase in the AR is expected to occur via the modification of PB domains by MPA shifting this energetic balance. The enhanced hydrophilicity of the MPA-modified PB block results in greater incompatibility with the PS block and lowered surface tension between the particle and surrounding, where both contribute to the particle elongation.

To further support this behavior, the control experiment using branched tetrathiol molecule, PETMP, was used for the selective modification of PB domains in PS_34k_-*b*-PB_25k_ particles (Figure S7). Similar to the modification with MPA, visual inspection showed PETMP-modified particles settled at the bottom upon centrifugation, similar to the observation in Figure S1. FT-IR spectra of PETMP-modified particles showed a clear reduction in the alkene =C–H bend near 908 cm^−1^ and the appearance of new peaks at 1722 cm^−1^ corresponding to the carbonyl (C=O) stretch and multiple new peaks in 1136–1231 cm^−1^ corresponding to the C–O stretch from the ester groups. However, no change in the AR of the particles was observed for PETMP-modified particles, in contrast to the case of MPA-modified particles. This result suggests that while functional thiols (MPA) graft onto the PB domain to mediate the interfacial interactions and affect the AR of ellipsoid, branched thiols rather crosslink the PB domain to fixate the particle structure.

**Figure 3:**
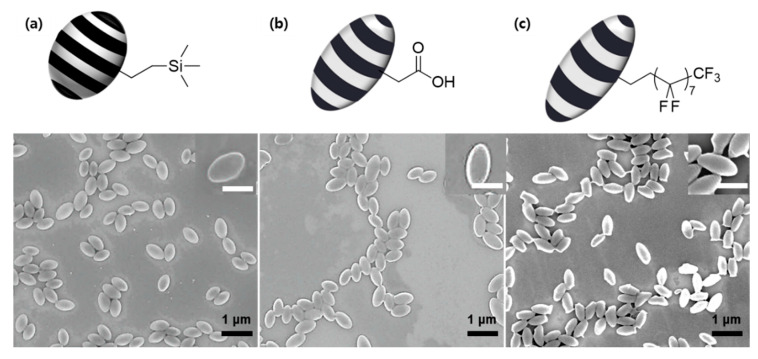
SEM images of PS_34k_-*b*-PB_25k_ particles that are modified using (**a**) mercaptoethyl)trimethylsilane (MTMS), (**b**) MPA, and (**c**) perfluorodecanethiol (PFDT). The feed ratio of thiols to double bonds was fixed to *r* = 0.5. Scale bars in the inset are 500 nm.

Based on the elongation behavior of PS_34k_-*b*-PB_25k_ particles upon the modification of PB domains, the versatility of the current approach was extended to examine the effect of functional thiols with different chemical structures on the particle shape. Three different functional thiols, MTMS, MPA, and PFDT were used for surface modification with the same feed ratio (*r* = 0.5) (Figure 3). Increase in the AR was observed for all three cases, demonstrating the generality of the surface modification approach for achieving particle elongation. Furthermore, the degree of particle elongation was dependent on the type of functional thiol, where the final AR was 1.58, 1.78 and 2.01 for MTMS, MPA, and PFDT-modified ellipsoids, respectively. Each particles had L = 575 ± 56 nm and S = 364 ± 97 nm for MTMS, L = 585 ± 51 nm and S = 328 ± 23 nm for MPA, L = 626 ± 43 nm and S = 312 ± 21 nm for PFDT. Assuming the interfacial energy between the two blocks constituted some of the driving force responsible for the elongation of the ellipsoid, segregation strength (χ) between the PS block and modified PB block plays important role in determining the AR. As thiols randomly react with double bonds to partially modify the PB domain, it is reasonable to regard the modified PB block as a statistical copolymer, similar to the example from Lodge and Hillmyer, where the effective segregation strength was estimated based on the binary interaction of polymer A and statistical copolymer (B-s-C) [55,56]. Therefore, the dissimilarity of the modified PB block and the PS block should determine the particle AR. The qualitative comparison of the polarity by adopting the solubility parameter (δ) for the functional groups (δ_PS_ = 18.6, δ_PB_ = 17.3 MPa^1/2^ and δ for poly(dimethylsilane), poly(acrylic acid), and poly(vinylidenefluoride) were adopted for δ_MTMS_ = 15.1, δ_MPA_ = 23.5, and δ_PFDT_ = 24.5) [57] shows that (δ_PS_- δ_PFDT_)^2^ > (δ_PS_- δ_MPA_)^2^ > (δ_PS_- δ_MTMS_)^2^. This supports that the greater elongation of ellipsoids is achieved by modifying with functional thiol has greater polarity (MTMS < MPA < PFDT), capturing the difference in experimental AR values for different functional thiols.

**Figure 4:**
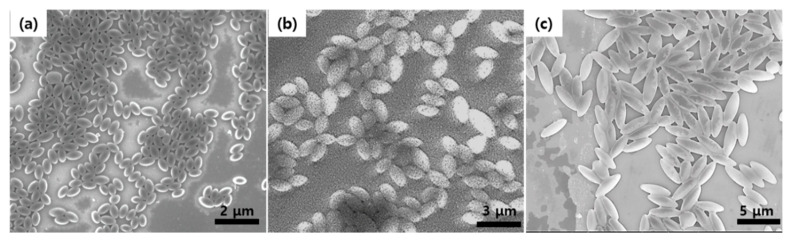
SEM images of the PS_34k_-*b*-PB_25k_ particles’ MPA modification (*r* = 0.5). Each particle was generated by membrane emulsification using different *d_pore_*, where pore sizes of (**a**) 0.5, (**b**) 1.1, and (**c**) 2.1 μm were used, respectively.

Another important parameter that provides the tunability of the AR of ellipsoids is the size of the particle. According to the mechanism of particle elongation, larger particles are easier to stretch since the surface energy of the droplets decrease relative to the bulk elastic and the interfacial free energy contributions on increasing the particle size. This strong influence of the particle volume on elongation is demonstrated by the significant increase in the AR along with the particle size (Figure 4). To achieve the controllability in the particle size, the pore size of the membrane used for emulsification was varied from 0.5, 1.1, to 2.1 μm as reported in our previous work [33,34,35]. Accordingly, the precursor particles before modification had AR values of 1.20 (*L* = 450 ± 45 nm and *S* = 376 ± 36 nm), 1.30 (*L* = 1166 ± 90 nm, *S* = 900 ± 65 nm), and 1.37 (*L* = 2037 ± 183 nm, *S* = 1488 ± 121 nm) for *d_pore_* = 0.5, 1.1, and 2.1 μm, respectively (Figure S8). Upon MPA modification, sharp increase in AR was observed for all cases where AR values were 1.78 (*L* = 585 ± 51 nm, *S* = 328 ± 23 nm), 1.94, (*L* = 1534 ± 168 nm, *S* = 794 ± 62 nm), and 3.07 (*L* = 3733 ± 272 nm, *S* = 1218 ± 77 nm) for 0.5, 1.1, and 2.1 μm, respectively.

**Figure 5:**
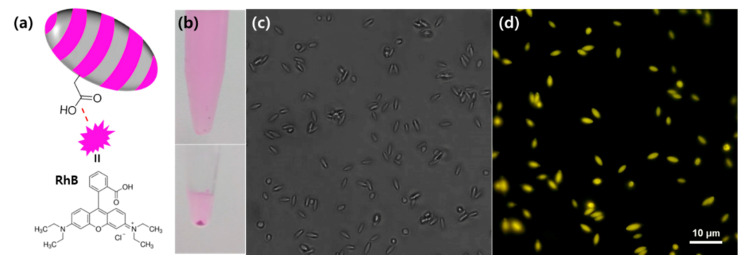
(**a**) Schematic showing the complexation of rhodamine B dye on the acid-functional surface of MPA-modified PS_34k_-*b*-PB_25k_ particles (*d_pore_* = 2.1 μm); (**b**) photograph of the dye-complexed particle before the (upper) and after the (bottom) centrifugation; (**c**) optical microscope; and (**d**) the fluorescent microscopic image of rhodamine B functionalized ellipsoidal particles.

Having the spatially controlled functionality on the surfaces of ellipsoids with stacked lamellae structure, the availability of the functional groups for the further complexation of functional molecules was investigated. Using the acid-functionalized particle obtained by MPA modification as an example (*L* = 3733 ± 272 nm, *S* = 1218 ± 77 nm, and AR = 3.0, produced by *d_pore_* = 2.1 μm), the post-functionalization of the ellipsoid surface with organic dye was achieved. Rhodamine B was chosen as a small molecule organic dye since it is a water-soluble and can effectively interact with surface-bound carboxylic acid groups via electrostatic interaction with ammonium chloride group (Figure 5a). To examine the functionalization, an aqueous suspension containing ellipsoids was incubated with an excess amount of rhodamine B and agitated for 1 h. Subsequently, the unbound dyes were removed by repeated centrifugation followed by decanting the supernatant where pure DI water was replenished for each cycle. Note that upon centrifuging the colored particle suspension, the ellipsoids were concentrated at the bottom of the tube and retained the color, which confirmed the rhodamine B dye bound to the ellipsoid particles (Figure 5b). To further confirm the dye complexed surface functionalization, particles were imaged with an optical microscope and fluorescence microscopy where ellipsoid particles exhibited the homogeneous fluorescent emission of yellow color, demonstrating the well dispersed and stable nature of the particles after dye complexation (Figure 5c,d). These functionalized particles show potential as a probe for orientation-dependent directional fluorescence.

## 4. Conclusions

The site-specific surface functionalization of a nanostructured PS-*b*-PB particle was demonstrated using photo-induced thiol-ene click reaction. The selective functionalization of PB domains on the particle surface was confirmed via ^1^H NMR and FT-IR, and the degree of PB modification was controllable by varying the feed ratio of thiols to the double bonds in the PB domain. In addition, the interesting elongation of the particle as a function of the degree of modification was discovered, due to the increased incompatibility between PS and modified PB block, and lowered the surface tension of the particles. Based on the particle elongation mechanism, the tunability of the AR of ellipsoids was expanded by controlling the particle size and chemical structure of functional thiols for modification, showing the generality of this modification approach. Finally, secondary functionalization on the particle surface via the electrostatic interaction of fluorescent dye allowed stable binding of rhodamine B on the well dispersed ellipsoidal particle. We envision that the surface functionalization of these nanostructured, nonspherical particles with specific interaction (e.g., hydrogen bonding) or stimuli-responsive groups can be utilized as new type of colloidal building block for constructing the colloidal superstructure.

## Figures and Tables

**Table 1:** The summary of average values of domain spacing (*L*_0_), domain thickness, major axis (*L*), minor axis (*S*), and AR (=*L*/*S*) for pristine PS_34k_-*b*-PB_25k_ particles and MPA-modified particles.

	*L*_0_ (nm)	*d*_*G*_ (nm)	*d*_*D*_ (nm)	*L* (nm)	*S* (nm)	AR
*r* = 0.0	33.7 ± 1.8	14.4 ± 1.3	18.9 ± 0.7	450 ± 45	376 ± 36	1.20
*r* = 0.1	36.9 ± 2.7	22.1 ± 1.2	16.1 ± 1.2	501 ± 40	320 ± 23	1.56
*r* = 0.2	38.0 ± 2.5	25.7 ± 1.9	14.0 ± 1.8	567 ± 56	322 ± 31	1.75
*r* = 0.5	39.6 ± 2.1	24.2 ± 1.7	14.0 ± 1.0	585 ± 51	328 ± 23	1.78

## Supplementary Materials

The following are available online at https://www.mdpi.com/2073-4360/12/12/2804/s1. Figure S1: Photograph of particles after centrifugation at 10,000 rpm for 5 min for (left) pristine PS34k-b-PB25k particles and (right) MPA-modified particles. While pristine PS_34k_-*b*-PB_25k_ particles floated on the top of the aqueous phase, MPA-modified particles were settled to the bottom. Figure S2: Magnified FT-IR spectra of PS_34k_-*b*-PB_25k_ particles modified with MPA for a varying degree of r = 0, 0.1, 0.2, 0.5. Normalization to C–H signal from PS block on 2800–3000 cm^−1^ shows a gradual increase in signal intensity near 1722 cm^−1^ corresponding to the carbonyl (C=O, purple) stretch from the carboxyl acid group. Table S1: Calculated conversion of PB modification for varying r. Figure S3: Full spectra of 1H NMR for MPA-modified PS_34k_-*b*-PB_25k_ particles with r = 0, 0.1, 0.2, and 0.5. Figure S4: TEM images of (a) before and (b) after MPA modification of onion-like PS_34k_-*b*-PB_25k_ particles. Figure S5: TEM image of MPA-modified PS_34k_-*b*-PB_25k_ particles treated with (a) OsO4 and (b) Au precursor (HAuCl4∙3H2O) before TEM imaging. Figure S6: SEM images of PS_34k_-*b*-PB_25k_ particles after reacting with MPA under UV irradiation for (a) 1 h, (b) 2 h, (c) 3 h. Figure S7: SEM image of (a) pristine PS_34k_-*b*-PB_25k_ particle and (b) PETMP-modified particle (r = 0.5). (c) FT-IR spectra comparing the pristine PS_34k_-*b*-PB_25k_ particle (black) and PETMP-modified particle (blue). (d) Schematic showing the crosslinking of PB domains via PETMP having branched tetrathiol groups. Figure S8: SEM images of PS_34k_-*b*-PB_25k_ particles produced by membrane emulsification using different d_pore_, where (a) 0.5, (b) 1.1, and (c) 2.1 μm of pore size was used respectively.
