# Supplementary material for: Effect of Site-Specific Functionalization on the Shape of Nonspherical Block Copolymer Particles
Source: Polymers (Basel). 2020 Nov 26;12(12):2804. doi: 10.3390/polym12122804 (PMC7760798; doi:10.3390/polym12122804)
Supplement: Supplementary file 1 [file polymers-12-02804-s001.pdf]

polymers

Supporting Information of

Effect of Site-Specific Functionalization on the Shape of Nonspherical Block Copolymer Particles

Jaeman J. Shin *

Department of Chemical and Biomolecular Engineering, Korea Advanced Institute of Science and Technology (KAIST), Daejeon 34141, Korea; jmshin90@gmail.com
* Correspondence: jmshin90@ucsb.edu

Received: 5 November 2020; Accepted: 24 November 2020; Published: date

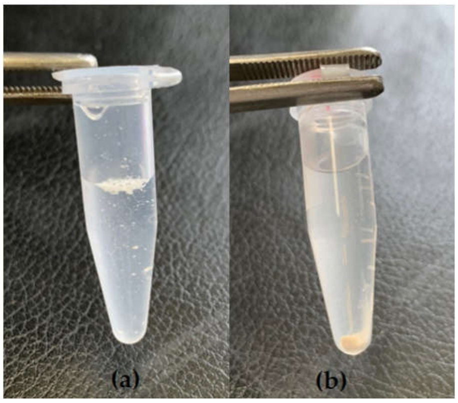

Figure S1. Photograph of particles after centrifugation at 10,000 rpm for 5 min for (**a**) pristine PS_{34k}-*b*-PB_{25k} particles and (**b**) MPA-modified particles. While pristine PS_{34k}-*b*-PB_{25k} particles floated on the top of the aqueous phase, MPA-modified particles were settled to the bottom.

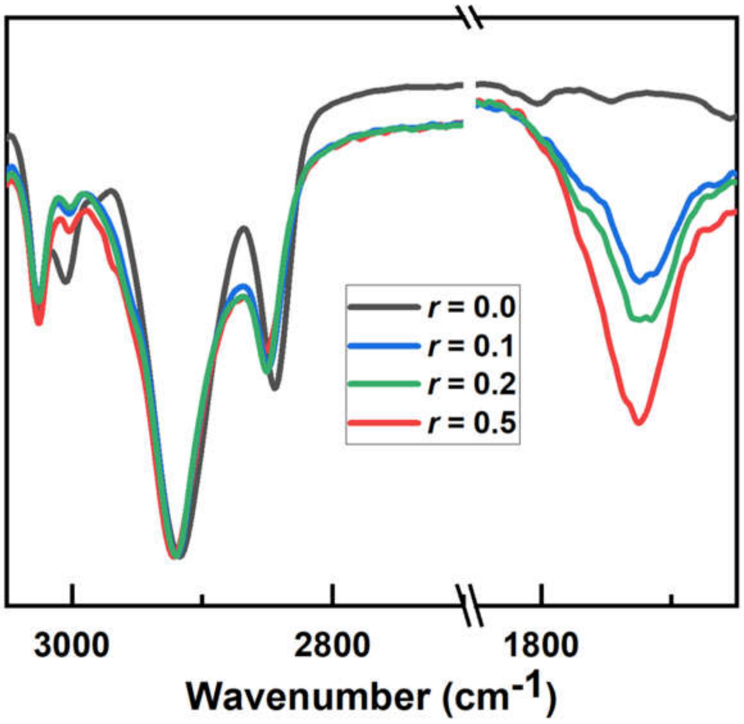

Figure S2. Magnified FT-IR spectra of PS_{34k}-*b*-PB_{25k} particles modified with MPA for a varying degree of r = 0, 0.1, 0.2, 0.5. Normalization to C–H signal from PS block on 2800–3000 cm^{-1} shows a gradual

increase in signal intensity near 1722 cm^{-1} corresponding to the carbonyl (C=O, purple) stretch from the carboxyl acid group.

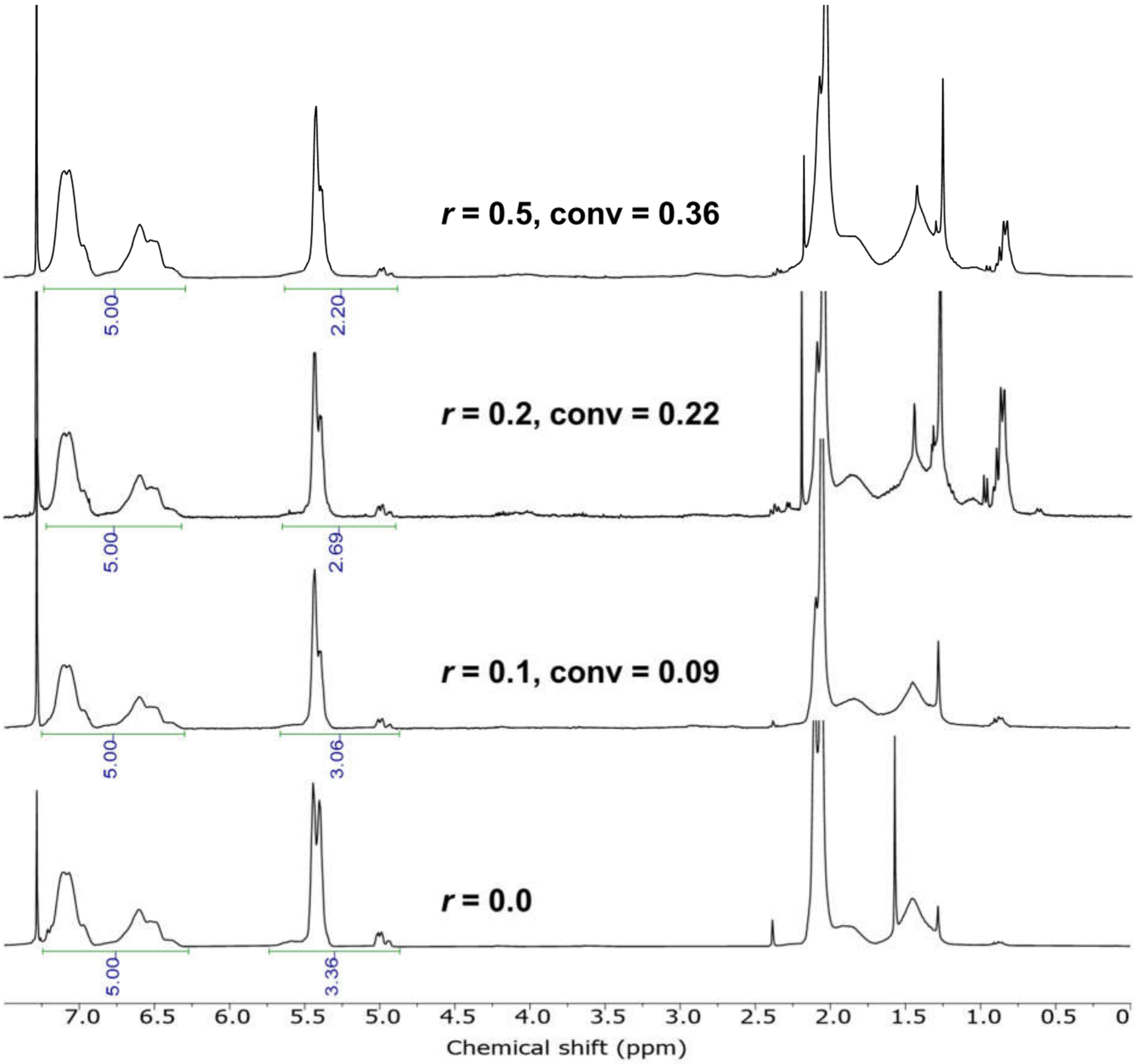

Figure S3. Full spectra of ^{1}H NMR for MPA-modified PS_{34k}-*b*-PB_{25k} particles with r = 0, 0.1, 0.2, and 0.5.

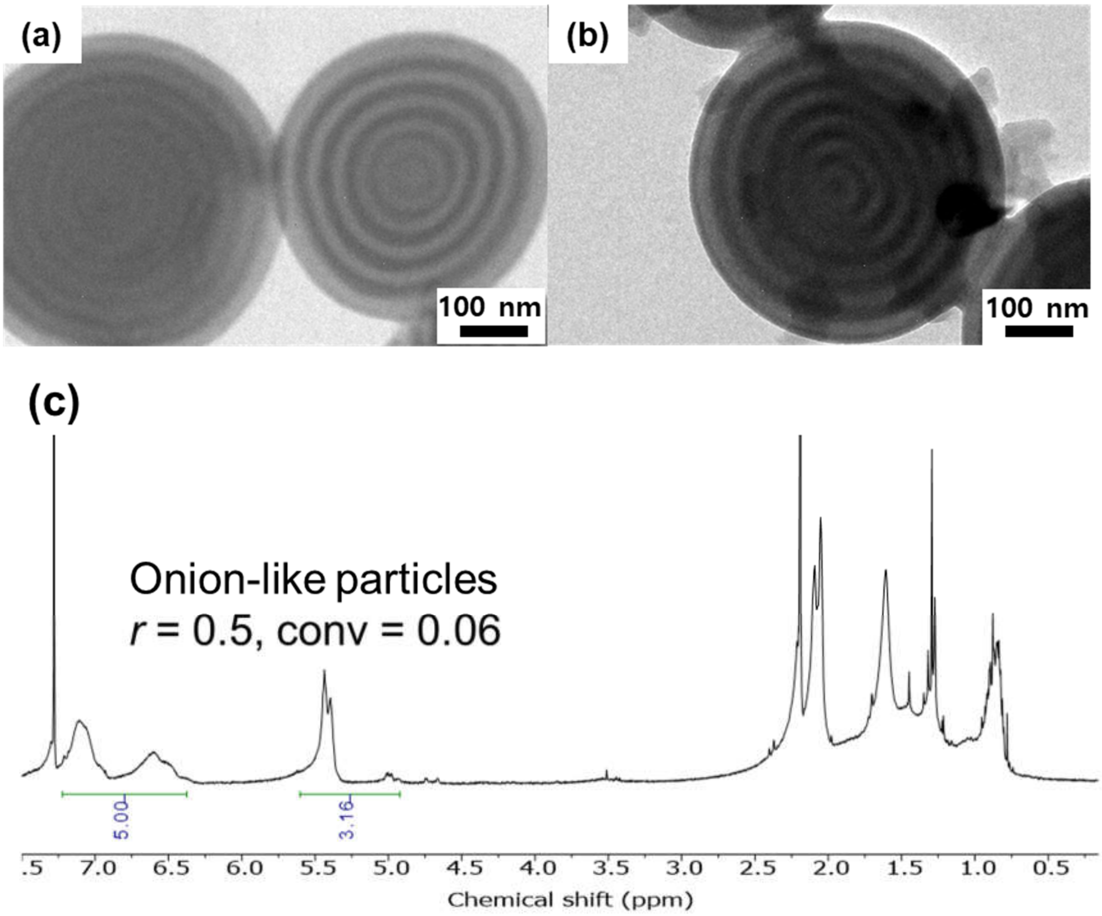

Figure S4. TEM images of (**a**) before and (**b**) after MPA modification of onion-like PS_{34k}-*b*-PB_{25k} particles, and (**c**) 1H NMR spectrum of modified onion-like particles showing minimal modification (low conversion of 0.06) even at high feed ratio of MPAs (*r* = 0.5).

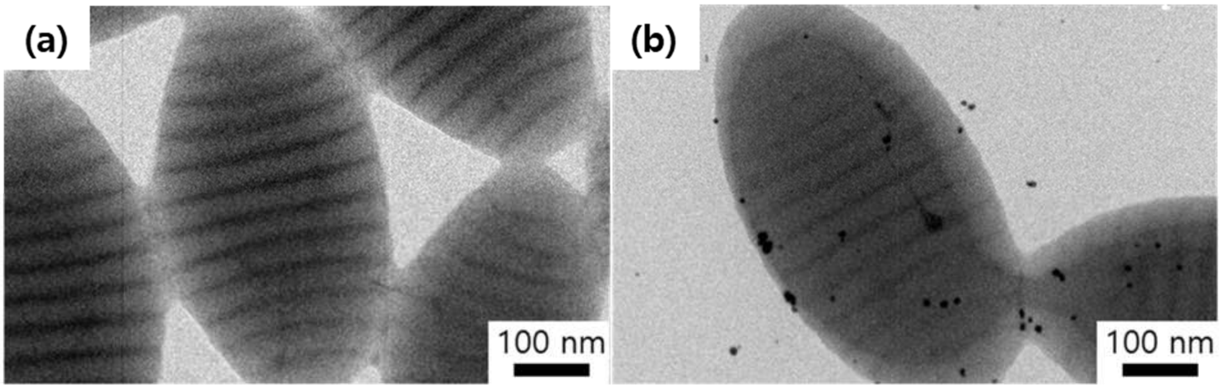

Figure S5. TEM image of MPA-modified PS_{34k}-*b*-PB_{25k} particles treated with (**a**) OsO_4 and (**b**) Au precursor ($HAuCl_4 \cdot 3H_2O$) before TEM imaging.

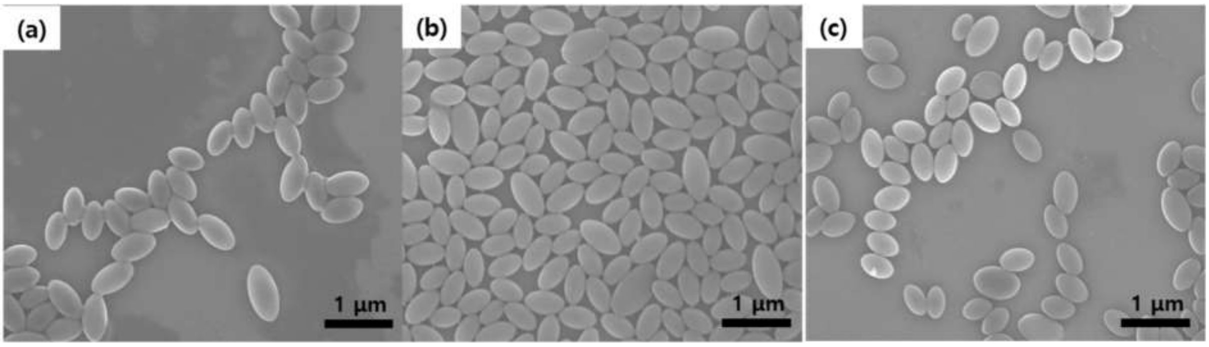

Figure S6. SEM images of PS$_{34k}$-*b*-PB$_{25k}$ particles after reacting with MPA under UV irradiation for (**a**) 1 h, (**b**) 2 h, (**c**) 3 h.

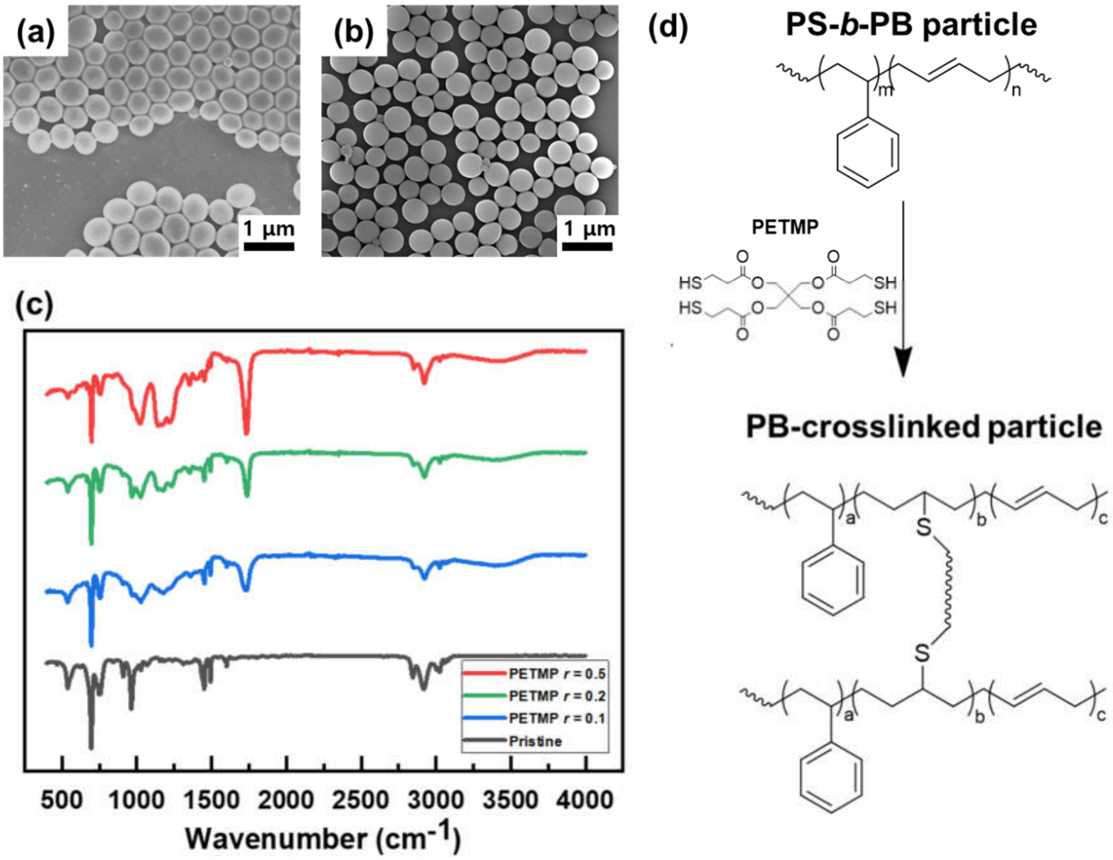

Figure S7. SEM image of (**a**) pristine PS$_{34k}$-*b*-PB$_{25k}$ particle and (**b**) PETMP-modified particle (r = 0.5). (**c**) FT-IR spectra comparing the pristine PS$_{34k}$-*b*-PB$_{25k}$ particle (black) and PETMP-modified particle (blue). (**d**) Schematic showing the crosslinking of PB domains via PETMP having branched tetrathiol groups.

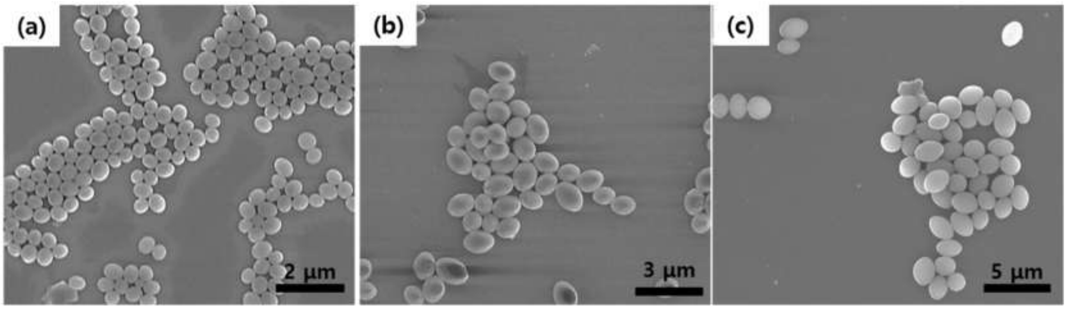

Figure S8. SEM images of PS$_{34k}$-*b*-PB$_{25k}$ particles produced by membrane emulsification using different d_{pore}, where (**a**) 0.5, (**b**) 1.1 and (**c**) 2.1 µm of pore size were used, respectively.
